# Concepts of epigenetics in prostate cancer development

**DOI:** 10.1038/sj.bjc.6604771

**Published:** 2008-11-11

**Authors:** C S Cooper, C S Foster

**Affiliations:** 1 Male Urological Cancer Research Centre, Institute of Cancer Research, 15 Cotswold Road, Sutton, Surrey SM2 5NG, UK; 2 Division of Cellular Pathology and Molecular Genetics, The University of Liverpool, Duncan Building, Daulby Street, Liverpool L69 3GA, UK

**Keywords:** epigenetic, prostate cancer, histone marks, DNA methylation, stem cells, small RNAs

## Abstract

Substantial evidence now supports the view that epigenetic changes have a role in the development of human prostate cancer. Analyses of the patterns of epigenetic alteration are providing important insights into the origin of this disease and have identified specific alterations that may serve as useful diagnostic and prognostic biomarkers. Examination of cancer methylation patterns supports a stem cell origin of prostate cancer. It is well established that methylation of *GSTpi* is a marker of prostate cancer, and global patterns of histone marking appear to be linked to cancer prognosis with levels of acetylated histones H3K9, H3K18, and H4K12, and of dimethylated H4R3 and H3K4, dividing low-grade prostate cancer (Gleason 6 or less) into two prognostically separate groups. Elevated levels of several components of the polycomb group protein complex, EZH2, BMI1, and RING1, can also act as biomarkers of poor clinical outcome. Many components of the epigenetic machinery, including histone deacetylase (whose expression level is linked to the *TMPRSS2:ERG* translocation) and the histone methylase EZH2, are potential therapeutic targets. The recent discovery of the role of small RNAs in governing the epigenetic status of individual genes offers exciting new possibilities in therapeutics and chemoprevention.

The term ‘epigenetic’ refers to mechanisms of inherited change of gene expression that do not involve changes in DNA sequence or copy number. Conventionally, this includes CpG methylation, histone modification or marking, and gene imprinting, but recent evidence shows that small RNAs could also play a critical role in directing epigenetic silencing. In cancers, when alterations in the normal epigenetic portrait arise, they are subjected to the same selective pressures as genetic alterations. However, epigenetic alterations have the potential to cause changes in the expression of individual or groups of genes at considerably elevated rates when compared with their genetic counterparts. A view is emerging that epigenetic disruption of progenitor cells may be an initial step in cancer development, leading to a polyclonal precursor population within which subsequent genetic and epigenetic events may occur. There is considerable interplay between different classes of epigenetic modification, but the mechanisms of interaction are poorly understood, and the hierarchical order of epigenetic alterations during unscheduled gene silencing in cancer is unknown. Here, following a summary of the evidence supporting the role of epigenetic change in prostate cancer development, we review new concepts and technologies emerging in this field that may have particular relevance to the clinical management of prostate cancer.

## Epigenetic changes in prostate cancer

### DNA methylation

The link between methylation at the N^5^-position of cytosine in CpG sequences and cancer development is well established. Cancer formation is accompanied by dramatic changes in the cellular methylation profile such that global demethylation of the genome occurs in parallel with CpG hypermethylation at specific genes strongly linked to their transcriptional inactivation. A list of genes that are hypermethylated at CpG islands in prostate cancer is shown in Table 1. Many of these genes, including *INK4a*, *RASSF1a* and *APC*, exhibit tumour suppressor functions whose inactivation associated with hypermethylation of CpG islands in 5′ regulatory regions occurs during cancer development. Inappropriate gene hypermethylation catalysed by DNA methylases (DNMTs) may represent an early event in cancer development, possibly linked to ageing. Methylation of *GSTpi* was absent in normal epithelium and present in 6.4% of proliferative inflammatory atrophy, in 70% of high-grade PIN and in 90% of prostate cancer (Nakayama *et al*, 2003). When methylation at the *APC* gene was considered together with methylation of *GSTpi*, the sensitivity for detecting cancer approached 100% (Jeronimo *et al*, 2004). Methylation may also be associated with tumour progression. For example, CpG hypermethylation of the cell adhesion gene *E-cadherin* in breast and prostate is integral to epithelial-to-mesenchymal transition that is believed to play a prominent role in tumour progression (Lombaerts *et al*, 2006). Methylation of the oestrogen receptor alpha (*ESR1*) gene, whose downregulation has been suggested to play a role in cancer metastasis, has also been documented in prostate cancer (Li *et al*, 2004). Hypomethylation of specific genes is also linked to prostate cancer development. For example, Wang *et al* (2007) have reported hypomethylation of *WNT5A*, *CRIP1*, and *S100P* in cancer but not in normal prostate. Interestingly, CpG methylation status appeared to control binding of MYB to the *WNT5A* promoter region.

### Histone marking

Histone alterations by methylation, acetylation, and ubiquitination (called ‘marks’) are inherited as epigenetic variations and linked to gene activation or silencing depending on the precise nature and position of the modification (called the histone code). Promoters of expressed genes are commonly associated with the active marks, such as H3 lysine 4 dimethylation (H3K4me2) and H3 lysine 9 acetylation (H3K9acetyl). In contrast, in transcriptionally silenced genes, these are replaced by repressive marks, including H3K27me3, H3K9me2, and H3K9me3. Changes in histone marks arise during cancer development and appear to have prognostic potential. Fraga *et al* (2005a) found that loss of monoacetylated lysine 16 and trimethylated lysine 20 forms of histone H4 is a global hallmark of human cancers, and in prostate cancer, global patterns of histone modification are linked to the risk of prostate cancer recurrence (Seligson *et al*, 2005). Particularly, the levels of acetylated histones at H3K9, H3K18, and H4K12, and of dimethylated H4R3 and H3K4, divided low-grade prostate cancer (Gleason 6 or less) into two prognostically separate groups.

Polycomb group (PcG) protein complexes PRC1–PRC4 play a key role in controlling transcriptional silencing. Polycomb group protein complex-1 contains BMI1 and RING1, whereas PRC2–PRC4 contain EZH2. Expression of all three proteins (BMI1, RING1, and EZH2) is associated with adverse pathological features in prostate cancer, but only BMI1 provides additional prognostic power in multivariate analysis. EZH2 is the component of the PRC2 complex responsible for catalysing methylation of both H3K27 and H1K26, but this protein can also become associated with DNMTs, and has a role in the induction and targeting of DNA methylation. Following the action of EZH2, the H3K27me3 mark attracts PRC1, which contains other proteins, including BMI1, which is involved in maintaining gene silencing. One of the target genes for EZH2 in prostate cancer cells is *DAB2IP* (Chen *et al*, 2005), whose encoded protein is a potent cell growth inhibitor and modulator of Ras-signalling. Overexpressed EZH2 becomes associated with the *DAB2IP* promoter and appears to facilitate recruitment of other components of EZH2 complex (Chen *et al*, 2005). These alterations are linked to increased levels of H3K27me2 and H3K27me3 and of histone deacetylase (HDAC)-1 associated with the promoter. The *MSMB* gene, which encodes the inhibitor of prostate cancer development PSP94, appears to be similarly targeted by EZH2 (Beke *et al*, 2007).

Formation of acetyl histone marks that are associated with transcriptional activation, is catalysed by histone acetyl transferases (HATs). In leukaemia, the HAT TIP60 directly interacts with, and is a co-activator for, the translocation activated transcription factor C/EBPa. The co-activator function is acetyltransferase dependent, with acetylation of histones 3 and 4 associated with activation of downstream genes. Histone acetyl transferases involved in histone marking may also acetylate non-histone proteins, although the relationship of these activities to epigenetic marking is unclear. For example, treatment of prostate cancers with di-hydrotestosterone induces acetylation at a lysine residue in the hinge region of the androgen receptor (AR) by the HATs p300, P/CAF, and TIP60. Acetylation enhances transactivation of the AR by coactivators (SRC1, Ubc9, and ARA70) and increases access to androgen-responsive elements. Similarly, TIP60 also has a role in modulating DNA repair: TIP60 acetylates p53, and activation of the DNA damage sensor ATM depends on TIP60. Haploinsufficiency of TIP60 has been linked to breast cancer development (Gorrini *et al*, 2007) and it would be interesting to see whether alterations in TIP60 are also important in the development of prostate cancer.

Acetyl groups are removed from both histone and non-histone proteins by HDACs. The *HDAC1* gene has been identified as a gene overexpressed in prostate cancers containing the *TMPRSS2–ERG* fusion (Iljin *et al*, 2006), with its highest levels found in hormone refractory disease. Another HDAC, SirT1, plays a role in transcriptional silencing of DNA hypermethylated cancer genes by localising to the gene promoters to deacetylate H4K16 and H3K9. Downregulation of SirT1 activity results in reactivation of silenced Wnt agonist genes (the SFRPs) and hence dampens signalling through the activated Wnt pathway in colon and breast cancer cells (Pruitt *et al*, 2006). These studies may also have relevance to prostate cancer where the Wnt pathway is known to be activated in a proportion of cases.

The relationship between DNA hypermethylation and polycomb-based histone modification appears complex. The conventional view (e.g., see discussion of ‘stem-cell-ness’ below) is that inactive histone marks are associated with DNA hypermethylated promoters, whereas active marks are normally associated with hypomethylated promoters. Recent data from Kondo *et al* (2008) challenge this view. Using a ChIP-based microarray approach, they found that in cancer around 5% of promoters (16% CpG, 84% non-CpG) were enriched with repressive mark H3K27me3. The genes containing this mark were specifically silenced in prostate cancer compared with normal tissue even though genes with CpG islands only showed low levels of DNA methylation. Downregulation of EZH2 and inhibition of deacetylases restored gene expression without altering promoter DNA methylation. This independence of DNA-methylation and histone marking appeared to conflict with previous studies but could be explained by tissue and cancer-specific mechanisms. Future studies will need to address why DNA methylation in cancer affects some silenced PcG targets but not others.

### Loss of imprinting

Imprinting is a process that allows specific expression from either the maternal or the paternal allele. Evidence suggesting a role for loss of imprinting in cancer development initially came from reports that loss of heterozygosity at the 11p15 locus in Wilms tumours invariably involved loss of the maternal allele and duplication of the paternal allele (Kurukuti *et al*, 2006). This chromosome region is now known to contain the reciprocally imprinted and adjacent *IGF-II* and *H19* alleles. These observations may be of particular relevance to prostate cancer because alterations both of insulin-like growth factors (IGFs) and of their binding proteins (IGFBPs) have been implicated in development of this disease. Biallelic expression of *IGF-II*, implying reactivation of the maternal allele, was observed in prostate cancers removed at radical surgery for localised adenocarcinoma as well as in samples of apparently normal adjacent tissue. In contrast, biallelic expression was absent from virtually all BPH specimens (Jarrard *et al*, 1995). DNA methylation of the imprinted suppressor gene *p57/Kip2* that encodes an embryonic cyclin-dependant kinase has also been found in a high proportion (56%) of prostate cancer (Lodygin *et al*, 2005).

A key question is whether the different classes of epigenetic changes discussed above are responsible for driving cancer development, or whether some of the alterations are a secondary consequence of the development of this disease. If the former is true, defects in the machinery controlling DNA-methylation and chromatin structure should be linked to cancer. This is indeed the case, although current supporting evidence is entirely restricted to cancer types other than prostate. It is not our intention to carry out a systematic review of this area apart from noting that mutations linked to cancer development in humans or mice have been found in genes encoding DNA methyltransferases, methyl-CpG-binding proteins, chromatin remodelling proteins (*SNF5*, *ATRX*), histone methyltransferase (*SUV39H*), histone acetylases (*MOZ*, *MORF*), and histone deacetylases (*HDAC2*).

## Rna-mediated transcriptional silencing

Small RNAs may have a key role in controlling the epigenetic state. It is well established that siRNAs can direct post-transcriptional silencing, but it is now becoming apparent that promoter-targeted siRNAs can also direct both the activation and repression of gene transcription through orchestrating epigenetic changes in a process that requires RNA polymerase II and Argonaute 1 and 2. The targeted promoter may be rendered transcriptionally silent, exhibiting the repressing marks H3K9me2 and H3K27me3 (Morris, 2008). Han *et al* (2007) provided evidence that a low-abundance mRNA containing an extended 5′-untranslated region that overlaps the gene promoter is required for RNA-directed epigenetic modifications. In their model, the low-copy-number RNAs transcribed by RNA polymerase II from the promoter region are recognised by an antisense siRNA, and function as a recognition motif to direct epigenetic silencing complexes to the corresponding target promoters. Human cells express many endogenous species of small RNAs, including endogenous siRNAs, that are candidates for involvement in this process. It is now important to establish whether this model represents a general mechanism of endogenous epigenetic control, and whether appropriately targeted siRNA can be used to permanently silence individual genes (e.g., the *TMRSS2–ERG* gene fusion) in novel therapeutic and chemopreventive approaches.

## Stem cell-ness in cancer development

**Figure 1:**
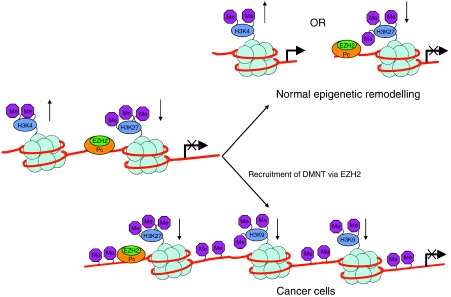
Working model for transition of bivalent stem cell chromatin to inactive chromatin during cancer development. In stem cells, chromatin exists in a bivalent state characterised by the absence of DNA methylation and the presence of both activating (e.g., H3K4me2) and inactivating (H3K27me3) markers. During cancer development, it is proposed that recruitment of DMNTs through EZH2 causes methylation of DNA. The inactive chromatin is characterised by the markers H3K9me2, H3K9me3, and H3K27me3. Model taken from the data presented by Ohm *et al* (2007) and Schlesinger *et al* (2007).

A popular view is that cancer stem cells or dividing precursor cells are the point of origin of individual cancers. In support of this hypothesis, cancers often exhibit alteration in pathways known to be involved in the preservation of stem cells. New evidence indicates that particular chromatin patterns controlling important regulatory genes in stem cells may leave these same genes vulnerable to DNA methylation during cancer development. Ohm *et al* (2007) compiled a set of 29 genes with tumour-suppressing potential that were frequently silenced by hypermethylation in cancer cells, including 16 that are hypermethylated in prostate cancer (Table 1). The authors found that these genes were usually unmethylated in both normal and malignant embryonic cells, but noted that most of these genes occurred within a subset (∼10% of all genes) associated with PcG in stem cells. Further analysis showed that, in embryonic stem (ES) cells, these genes are held in a ‘bivalent’ transcription-ready state by a promoter chromatin pattern consisting of the repressive mark histone H3K27me3 produced by PcG proteins, including SUZ12, EZH2, and SirT1, and by the active mark H3K4me2 (Figure 1). In embryonic carcinoma cells, two additional repressive marks, dimethylated H3K9 and trimethylated H3K9, which are each associated with DNA hypermethylation in adult cancers, were also present.

Direct support for the idea that genes held in this ‘bivalent’ state are susceptible to methylation was provided by analyses of individual clones of stem cells containing ectopically overexpressed BMI1 (BMI1 is a central component of PRC1 involved in recognition of the H3K27me3 mark, established by EZH2, and has a role in subsequent maintenance of PcG-mediated long-term gene silencing). Clones of stem cells were identified that exhibited increased methylation of the Wnt antagonist gene *SFRP5*, and where the pattern of histone methylation had changed to resemble more closely that found in cancer cells.

Schlesinger *et al* (2007) demonstrated that genes methylated in cancer cells are specifically packaged with nucleosomes containing H3K27me3. This chromatin mark is established on unmethylated CpG island genes early in development and then maintained in differentiated cell types by the presence of an EZH2-containing P complex. In cancer cells, the presence of this complex brings about the recruitment of DNA methyltransferases, leading to *de novo* methylation (Figure 1). These results also suggested that the tumour-specific targeting of *de novo* methylation is pre-programmed by an established epigenetic system that normally has a role in marking embryonic genes for repression.

## An epigenetic link to cancer aetiology

It is thought that lifestyle and environmental or dietary exposure may contribute to cancer risk through epigenetic mechanisms. Although little data relate directly to prostate cancer, examination of other systems provides strong evidence that the occurrence of epigenetic changes is age related. Ageing is associated with global hypomethylation together with hypermethylation of specific genes, in the same way as in cancer. Inactivation of specific genes by DNA methylation in the ageing colonic mucosa has been proposed to be one of the earliest events in the development of cancer at this site (Issa *et al*, 1994). Analysis of monozygotic (identical) twins demonstrate that they are epigenetically indistinguishable in early life. However with ageing, differences in DNA methylation patterns, histone modification and gene expression pattern are observed. These differences were most pronounced when the twins had lived apart, suggesting a role for diet and environment (Fraga *et al*, 2005b). SirT1 and IGF signalling, both linked to epigenetic mechanisms, have each been implicated in controlling the ageing process.

A variety of dietary and environmental factors have been linked to epigenetic changes: these include smoking, alcohol consumption, drinking green tea, dietary selenium levels, folate and methionine deficiencies, and the presence of resversatrol and dihydrocoumarins; high selenium consumption is known to be associated with lower risk of developing prostate cancer. Resversatrol (a molecule present in several plants including purple grapes) and dihydrocoumarins (found in sweet clover) inhibit SirT1. Prostate cancer frequently progresses very slowly, and for many cases, treatments that delay the development or progression of this disease, even marginally, could have a significant clinical impact. If epigenetic alterations occur gradually through ageing or under the influence of dietary factors as the first step in cancer development, they may provide an excellent target for preventative strategies. In this respect, it is worthy of note that the potential chemopreventive agents sulphoraphane, butyrate, and diallyl disulphide are all known to act as inhibitors of HDACs. The antioxidant EGCG, present in green tea, is a DNMT inhibitor. Support for the idea that chemoprevention strategies can be successful comes from the study of patients with genetically determined hyperhomocysteinemia where treatment by folate supplementation can restore the normal DNA methylation and expression patterns of specific genes (Ingrosso *et al*, 2003).

Epigenetic marks may not always be erased by passage through the germ line, and in some cases can be inherited from one generation to the next. The influence of maternal diet in this process is well documented. It is, however, now emerging that paternal transmission may also occur. This has been described in mice for the *Axin*(*Fu*) allele, and epidemiological evidence (Pembrey *et al*, 2006) supports the view that epigenetic effects of smoking can be inherited thorough father-to-son transmission. The general failure to identify gene mutations that account for genetic inheritance in prostate cancer suggests that it may be appropriate to examine epigenetic mechanism of inheritance. The identification of such genes represents a significant technical challenge, although technologies for the genome-wide screening of DNA-methylation patterns and histone marks that would be required for target gene identification are starting to become available (see below).

## Genome-wide analysis of epigenetic patterns

Whole genome analysis of histone modifications and of proteins involved in epigenetic modulation can be performed using ChiP-based approaches (Zhao *et al*, 2007; Roh and Zhao, 2008). For example, Zhao *et al* (2007) used ChiP coupled to a sequencing strategy to explore H3Kme3 and H3K27me3 landscapes in human ES cells. A variety of strategies involving high-throughput DNA sequencing or hybridisation to oligonucleotide micorarray may be used for the genome-wide analysis of DNA methylation pattern (reviewed by Zilberman and Henikoff, 2007). For example, Schumacher *et al* (2006) analysed enriched unmethylated DNA from human brain or Affymerix tiling arrays for chromosomes 21 and 22 and found, as expected, that most unmethylated sites were close to the 5′-end of genes. Using SNP arrays at 50 k and 250 k resolution (Kerkel *et al*, 2008) has demonstrated that genome-wide approaches can be used also to identify allele-specific methylation. The specific applications of these technologies to prostate cancer represent an important area of future investigation.

## Epigenetics and cancer therapy

Enzymes controlling epigenetic status and involved in cross talk between epigenetic systems are potential targets for cancer therapy. The enzymes include HDACs, HATs, co-activators including TIP60, PcG proteins including EZH2, and the DNMTs. In principle, enzymes that modulate the activities of these proteins are also potential drug targets, as are proteins that bind methylated CpG sequences. One strategy would be to try to use inhibitors of these proteins to reprogramme cells returning their epigenetic status to that reminiscent of a normal cell. A complication with this targeting strategy is that the effects of each individual targeted protein are most likely to be complex and difficult to predict. Several HDAC inhibitors have activity in the nanomolar range and have been demonstrated to inhibit growth of prostate cancer. Clinical trials have, however, been largely restricted to cancer types other than prostate (Gallinari *et al*, 2007). Such drugs may have relevance to cancers containing the *TMPRSS2:ERG* fusion where *HDAC1* is overexpressed (Iljin *et al*, 2006). In this respect, romidepsin, a bicyclic depsipeptide HDAC inhibitor, has a disease control rate of 14% when used to treat patients with hormone refractory disease (Parker *et al*, 2007).The ability of 5-azacytidine and other nucleoside analogues to inhibit DNMTs, cause hypomethylation and alter gene transcription in cultured prostate cancer cells is well documented, but only limited clinical trials have been performed.

## Conclusion

Epigenetics is now accepted as mainstream area of cancer research. However, many challenges remain both in understanding its importance in cancer development and in applying new knowledge to the benefit of prostate cancer patients. There is an urgent need to comprehensively assess epigenetic alterations on a genomic scale in a broad variety of normal cells, stem cells, and in corresponding cancer cells and to assess the importance of the role of small RNAs and their therapeutic potential.

## Figures and Tables

**Table 1:** Genes showing frequent hypermethylation in human prostate cancer

**Gene**	**Hormone receptors**	**Cell-cycle control**	**Repair or avoidance of DNA damage**	**Signal transduction**	**Cell adhesion and basement membrane**	**Inflammation response**	**Suppressor or candidate tumour suppressor**	**Unmethylated in human ES cells (Ohm *et al*, 2007)**
*APC*							√	√
*RBP1 (CRBP1)*							√	√
*CAV1*								
*CCND2 (cyclin D)*		√					√	√
*CD44*					√			
*CDH2*					√			√
*CDKN2A (INK4a)*		√					√	√
*DAB2IP*				√			√	
*DAPK*				√			√	√
*EDNRB*				√				
*ESR1*	√							
*ESR2*	√							
*FHIT*							√	
*GSTP2*			√				√	√
*HIC2*							√	√
*LAMA3*					√			
*LABM3*					√			
*MDR1*			√					
*0* ^*6−*^ *MGMT*			√				√	√
*PTGS2*						√		
*RAR-β*	√						√	√
*RASSF1*				√			√	√
*SFPR1*							√	√
*TIMP-3*							√	√
*TMS-1 (PYCARD)*							√	√

ES=embryonic stem.

The gene functions are shown. Many have tumour suppressor or potential tumour suppressor functions, and those genes shown by Ohm *et al* (2007) to be unmethylated in human ES cells are shown.
